# Comparative Forced Degradation Study of Anticomplement C5 Biosimilar and Originator Monoclonal Antibodies

**DOI:** 10.3390/ph18040579

**Published:** 2025-04-16

**Authors:** Merve Celik Yamaci, Ceren Pamukcu, Yigit Erdemgil, Ahmet Emin Atik, Zeynep Zulfiye Yildirim Keles, Ozge Can

**Affiliations:** 1 Department of Medical Biotechnology, Institute of Health Sciences, Acibadem Mehmet Ali Aydinlar University, 34638 Istanbul, Türkiye; 2 Biotechnology Group, Turgut Ilaclari A.S., 41400 Kocaeli, Türkiye; 3 Department of Natural Sciences, Faculty of Engineering and Natural Sciences, Acibadem Mehmet Ali Aydinlar University, 34638 Istanbul, Türkiye; 4 Department of Biomedical Engineering, Faculty of Engineering and Natural Sciences, Acibadem Mehmet Ali Aydinlar University, 34638 Istanbul, Türkiye

**Keywords:** monoclonal antibody, biosimilar mAb, forced degradation, size exclusion chromatography, isoelectric focusing, capillary electrophoresis, biological activity

## Abstract

**Background/Objectives**: The stress testing of biotherapeutic products is a critical component of drug development, enabling the assessment of stability, biosimilarity, and degradation pathways. Subjecting biosimilar monoclonal antibodies to controlled stress conditions yields essential insights into their structural and functional integrity, informing formulation optimization and mitigating risks before clinical trials. In this study, biosimilar products were comprehensively characterized and compared with originator products under forced degradation. The aim was to expose the products to different stress conditions such as oxidative, pH, thermal, freeze/thaw, and agitation. The products were then tested at defined time points using validated analytical methods. **Methods**: This study employed size-exclusion chromatography to detect aggregated forms. Isoelectric focusing characterized protein charge variants (e.g., acidic/basic isoforms) from post-translational modifications, while capillary electrophoresis quantified product-related impurities (aggregates and fragments). In addition, a complement assay was used to determine the efficacy and potency under specific stress conditions. **Results**: Our findings showed that biosimilar and originator products exhibited similar degradation profiles. The biosimilar monoclonal antibody was found to be analytically similar to the originator product in terms of critical parameters related to efficacy and safety under various stress conditions such as aggregation profile, biological activity, and charge variant distribution. **Conclusions**: Forced degradation studies facilitated the comprehensive and well-validated characterization of the structure and biological activity of biosimilar monoclonal antibody products.

## 1. Introduction

Technological advancements have dramatically boosted biotech drugs’ value, making them the world’s dominant pharmaceutical class through enhanced development and production capabilities [1,2]. These drugs have facilitated notable progress in the treatment of various diseases such as cancer and autoimmune diseases. Given the considerable impact of biotechnology on the global economy and society, prominent pharmaceutical companies have embraced biotech methods to introduce novel pharmaceutical products [2,3]. Biotech drugs, also known as biopharmaceuticals, comprise a high portion of the new drugs developed to treat various diseases, including cancer, cardiovascular disease, diabetes, inflammation, and autoimmune disorders [4]. The growing prevalence of approved monoclonal antibodies (mAbs) in the biopharmaceutical industry and their dominance among the top 10 best-selling drugs indicates the increasing significance of mAbs [5,6].

mAbs have transformed modern medicine by offering highly specific and effective treatments for diseases. They play a crucial role in therapeutic and diagnostic applications because of their ability to selectively target disease-associated antigens with high affinity and specificity. While mAbs offer therapeutic benefits, they encounter challenges like instability, difficult delivery, and immunogenicity that limit their clinical application. Because mAbs require complex biotechnological procedures such as mammalian cell culture, purification, and rigorous quality control measures, high production costs remain the primary challenge [7,8,9].

Exorbitant clinical trial costs challenge biopharmaceutical companies to develop affordable yet highly effective medications. Patent expirations have spurred a biosimilars market, increasing the affordability of critical biopharmaceuticals like mAbs [10,11]. Notably, the approval of a biosimilar product depends on its demonstrated comparability to the originator product in terms of quality, safety, and efficacy [9,12,13]. Despite adherence to the same protocols employed in the originator product, variations in manufacturing, storage, and handling may affect biosimilar mAb quality [14]. Therefore, biosimilar development necessitates comprehensive physicochemical and biological characterization using advanced analytical methods. This enables the precise identification of structural differences and a demonstration of similarity to the originator product [15,16].

mAbs are often exposed to various environmental stress conditions (e.g., heat, oxidation, agitation, and freeze–thaw cycles) during manufacturing, storage, transport, and administration. These conditions can markedly affect the quality of mAbs and, consequently, their therapeutic efficacy. To maintain consistent shelf-life performance, manufacturers must understand how mAbs degrade under different conditions. Forced degradation studies support mAb formulation development and lifecycle consistency through systematic stability investigations [17]. Despite being conducted under harsh conditions within a short period, these studies are capable of mimicking the accidental exposure of mAbs to conditions that could potentially impact product quality, safety, and effectiveness during manufacturing, storage, transportation, and administration [18,19].

In this study, the biosimilar and originator mAbs were thoroughly characterized using multiple analytical techniques and comparatively analyzed under forced degradation studies. mAbs were exposed to different stress conditions such as oxidative, pH, thermal, freeze/thaw, and agitation. The products were then tested at defined time points using validated analytical methods. These methods aimed to detect aggregated forms using size exclusion chromatography (SE-UPLC), charge variants using isoelectric focusing (icIEF), and purity/impurity levels using capillary electrophoresis (CE-SDS). In addition, a complement assay was utilized to determine potency under certain stress conditions.

## 2. Results and Discussion

All stress results for biosimilar mAbs (BS) are summarized in Table 1 and for the originator mAb (OR) in Table 2.

### 2.1. Thermal Stress

Thermal stress studies typically involve experiments conducted at two specific temperatures. The ideal condition for stress studies is a temperature of 37 °C, at which samples are treated with enzymes [16,20]. The second temperature is intentionally selected to exceed the degradation threshold of the products within the desired time frame, preventing the molecules from reaching their respective melting temperatures [21,22].

The Anti-C5 antibody biosimilars and their originators (EU and USA series) possess melting temperatures within 70.0–70.2 °C [23]. Stress administration at temperatures 10–20 °C below melting temperature values is recommended to account for the amplified molecular impact near thermal transition points [16,24]. As the applied temperature in thermal stress approaches the melting temperature, the mAb structure begins to unfold, resulting in accumulated opened intermediates. This phenomenon may elevate aggregation levels through self-aggregation or degradation [25,26]. Consequently, to enhance the comprehension of thermal stress, the second thermal stress condition was designated as 50 °C.

The samples were subjected to thermal stress by incubating them for 3, 7, and 14 days at 37 °C and 50 °C. The samples taken from the incubation at the specified points were stored at 5.0 ± 3.0 °C until analysis. Analyses using SE-UPLC, rCE-SDS, nrCE-SDS, and icIEF were performed to assess the effects of thermal stress on these samples. More details about the thermal stress were revealed by the complement assay analysis for potency effects.

**Figure 1:**
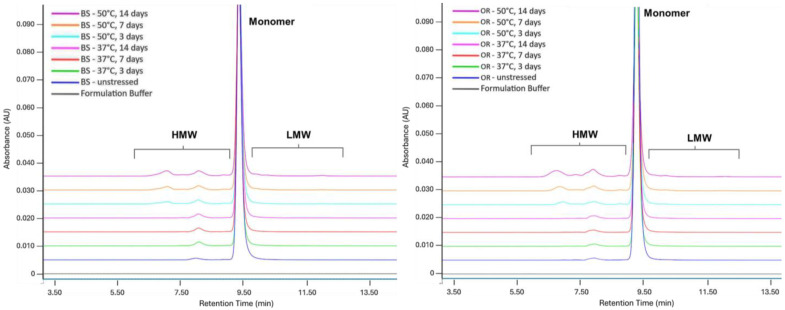
SE-UPLC chromatograms of BS (**left**) and OR (**right**) subjected to thermal stress at 37 °C and 50 °C for 3, 7, and 14 days. Samples were analyzed at a concentration of 2.5 mg/mL.

SEC results show the change in BS and OR monomer and aggregation (HMW) levels when stored for 3, 7, and 14 days at 37 °C and 50 °C relative to their controls at 5.0 ± 3.0 °C. In the BS and OR samples incubated at 37 °C for 14 days, monomer levels decreased by 1.3% and 1.2%, respectively, while HMW levels increased by 1.2% and 1.2%. In the chromatograms of all samples at 50 °C, monomer levels decreased visibly, while HMW levels increased. OR showed the greatest loss in the monomer level (11.6%) when incubated at 50 °C for 14 days. Both the BS and OR samples showed a higher tendency to aggregate (HMW) than the fragment (LMW). As expected, BS and OR samples showed more significant monomer degradation after 50 °C thermal stress. When linear curves were used to compare the degradation of monomer levels, a significant difference existed between the linear curves at 50 °C, where degradation was more pronounced (*p* < 0.0001). The overlay chromatograms are given in Figure 1.

**Figure 2:**
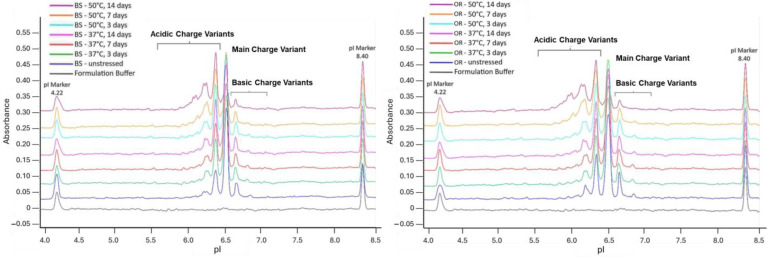
icIEF electropherogram of BS (**left**) and OR (**right**) subjected to thermal stress at 37 °C and 50 °C for 3, 7, and 14 days. Samples were analyzed at a concentration of 3.0 mg/mL.

After incubation at 37 °C and 50 °C, when the changes in charge variants due to thermal stress were examined, the levels of the acidic peaks for BS and OR increased while the levels of the main charge variants for BS and OR decreased. Following a 14-day incubation period, the main charge variant levels of the BS and OR decreased by 9.8% and 9.3% at 37 °C and by 34.5% and 33.1% at 50 °C, respectively. Notably, the main charge variant’s decrease resulted from the increased acidic charge variants. The acidic charge variants of BS and OR increased by 11.5% and 10.8% at 37 °C and 39.0% and 38.7% at 50 °C, respectively, after 14 days of incubation. The observed increase in acidic variants is consistent with previous studies showing that thermal stress promotes post-translational modifications such as the deamidation of asparagine, the formation of pyroglutamate, and the truncation of C-terminal lysine [16]. The deamidation of asparagine residues introduces additional negative charges and leads to an increased proportion of acidic charge variants. Similarly, the removal of the basic lysine residue at the C-terminal end of the protein reduces the number of basic charge variants and results in a relative increase in acidic variants or main species. These changes directly correlate with the changes observed in charge variant profiles during forced degradation studies. The basic charge variants were observed to have decreased by 1.7% and 1.6% at 37 °C and by 4.4% and 5.6% at 50 °C following the 14-day incubation of BS and OR. The rates of charge variant change in both temperature conditions are statistically similar, by comparing the slopes of the linear curves corresponding to main, acidic, and basic charge variant changes. The overlay electropherograms are given in Figure 2. Since the deamidation of asparagine residues in complementarity-determining regions decreases antigen-binding affinity, increased acidic charge variants may decrease mAb efficacy. Studies have suggested that deamidation and C-terminal Lys clipping are non-critical modifications that often do not affect the binding affinity, potency, or safety profile of mAbs, especially when detected in non-CDR regions [27,28]. Nevertheless, since the C-terminal Lys, which influences the presence of basic charge variants, is quickly eliminated by proteases following administration, basic charge variants normally have little effect on mAb safety or effectiveness [29].

**Figure 3:**
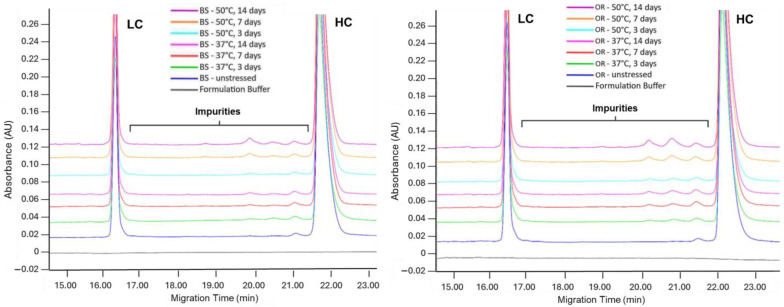
Reduced CE-SDS electrograms of BS (**left**) and OR (**right**) subjected to thermal stress at 37 °C and 50 °C for 3, 7, and 14 days. Samples were analyzed at a concentration of 1.0 mg/mL.

The LC + HC value obtained under reducing CE-SDS conditions gives the purity level, and the remaining values give the impurity level. Under both temperature conditions, the LC + HC value decreased significantly in the OR sample at the end of the 7th day and in both the BS and OR samples at the end of the 14th day compared to the control samples (*p* = 0.01 and *p* < 0.0001, respectively). On the 14th day of incubation at 50 °C, the decrease in the LC + HC levels of BS and OR samples was 7.2% and 7.9%, respectively. When LC + HC levels were compared using linear curves, a significant difference between the linear curves was observed at 50 °C, where the impurity levels in the samples increased proportionally with time (*p* = 0.02). One of the stress conditions in which the HMW rate rises the highest is thermal stress application [18]. Nevertheless, rCE-SDS analysis revealed no variant with a higher molecular weight than HC. The lack of higher molecular weight species in rCE-SDS indicates that the aggregates identified in SE-UPLC analysis are noncovalently bonded, consistent with previous studies on mAb degradation [30]. The overlay electrograms are given in Figure 3.

**Figure 4:**
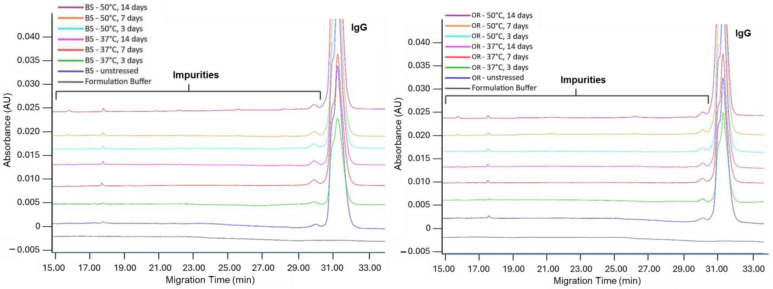
Non-reduced CE-SDS electrograms of BS (**left**) and OR (**right**) subjected to thermal stress at 37 °C and 50 °C for 3, 7, and 14 days. Samples were analyzed at a concentration of 1.0 mg/mL.

When IgG levels determined by nrCE-SDS analysis were compared with control sample levels, a significant difference existed in the OR sample only after 14 days of incubation at 50 °C (*p* = 0.001). After 14 days of incubation, the differences between samples and controls were 1.6% for BS and 1.1% for OR at 37 °C and 1.3% for BS and 1.7% for OR at 50 °C. No significant slope difference was observed between the two temperature conditions when linear curves were used to compare IgG levels. The overlay electrograms are given in Figure 4.

Relative potencies were only assessed for samples incubated for 14 days at 37 °C and 50 °C as these were the most severe conditions. The percentage of the relative potency of the thermally stressed BS and OR was calculated against the unstressed control samples. The relative potency results for the BS and OR samples showed similar results to each other under the same temperature conditions. In the 37 °C temperature condition, the relative potency results for BS decreased to 85.8% and for OR to 84.0%. In the 50 °C temperature condition, the results decreased to 80.4% for BS and 77.7% for OR. The biological activity decreased when the temperature increased from 37 °C to 50 °C. These results align with previous research on biosimilar and originator mAbs, indicating that enhanced aggregation and charge variations resulted in reduced antigen-binding efficacy. Although acidic variants can reduce binding affinity, the impact on clinical efficacy depends on the specific location of the modifications and their influence on Fc effector function [31]. Despite elevated aggregation and acidic variants in both BS and OR, complement assay results maintained acceptable range compliance.

### 2.2. Agitation Stress

Agitation stress is a notable physical stress condition that mAbs encounter during various stages, including purification, formulation, filtration, filling, finishing, shipping, and handling [32]. This stress condition serves as an effective means to monitor and assess the stability of biopharmaceutical drug formulations, as it can accelerate the formation of both insoluble and soluble aggregates. Polysorbate, a nonionic surfactant, accumulates at the air–liquid interface and is therefore widely used in mAb formulations to prevent mechanically induced aggregation. Notably, a 0.005% *w/v* polysorbate concentration prevents aggregate formation [33,34,35].

After subjecting the samples to 400 rpm agitation for 24 h and 72 h, the following changes were observed: In the BS samples, the monomer level decreased by 0.63% after 24 h and by 0.66% after 72 h, while the aggregate level increased by 0.69% and 0.71%, respectively. In the OR samples, the monomer level decreased by 0.48% after 24 h and by 0.52% after 72 h, with the aggregate level increasing by 0.51% and 0.53%, respectively. A comparison of the linear curves for the BS and OR samples revealed no significant difference between the slopes (*p* = 0.48 for the monomer and *p* = 0.43 for aggregation).

After 24 and 72 h of agitation stress, a slight decrease was observed in the main charge variants, while an increase was observed in the acidic and basic charge variants. A comparison of the charge variant levels of BS and OR with control samples at each time point showed no significant differences. Oxidation is a known degradation pathway under agitation stress beyond aggregation. Liquid–air and liquid–container interfaces can cause the oxidation of mAbs, which might alter their charge heterogeneity and perhaps cause them to lose their function. However, our results showed no significant differences in charge variant profiles between stressed and control samples, consistent with previous research showing minimal changes in charge heterogeneity under similar conditions [33,34]. The slight decrease in main charge variants was accompanied by increased acidic and basic charge variants, indicating minor structural changes but not at levels that would affect function.

At the end of 24 and 72 h of agitation stress, LC + HC levels decreased by 1.0% and 1.1% in BS and 0.94% and 1.1% in OR, respectively, compared to the controls. The decrease in IgG levels for BS and OR was 0.34% and 0.56% after 24 h and 0.55% and 0.79% after 72 h, respectively.

Although this study provides valuable information on the effects of agitation on mAbs, some limitations remain. First, the study was conducted under controlled laboratory conditions that may not fully mimic the dynamic stresses encountered during real-life production, transport, and storage. Second, the study focused mainly on physical degradative pathways, and further work is needed to investigate potential biochemical modifications such as oxidation and deamidation using advanced mass spectrometry techniques. In addition, the clinical significance of these findings will be further elucidated by functional assays assessing antigen-binding activity and effector functions under prolonged conditions.

### 2.3. pH Stress

Environmental pH influences mAb stability. When exposed to extremely acidic or alkaline conditions, mAbs may aggregate or become permanently denatured due to the disruption of the intermolecular and intramolecular forces that maintain their structure [21,36,37]. Applying pH stress can trigger degradations such as aggregation, fragmentation, and deamidation, which are accompanied by higher-order structural changes and a loss of bioactivity [18]. Additionally, selecting a pH close to the isoelectric point (pI) can create solubility issues, complicating the interpretation of degradation data [20]. However, to avoid thermal stress, which complicates the degradation pathways associated with pH stress, conducting these studies at room temperature is recommended [22].

**Figure 5:**
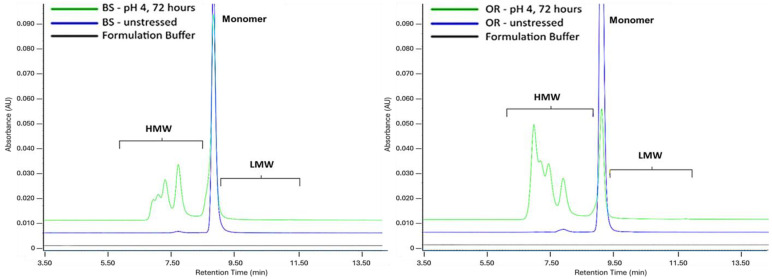
SE-UPLC chromatograms of BS (**left**) and OR (**right**) subjected to pH 4 acidic stress for 72 h. Samples were analyzed at a concentration of 2.5 mg/mL.

The acidic pH stress condition was established as the BS product being incubated for 72 h at pH 4, which is nearly the lowest pH value attained during the purification stages. The monomer levels of the OR and BS samples decreased to an extremely low level and the aggregate levels increased after 72 h of incubation at pH 4. Acidic pH 4 incubation caused 44.4% (BS) and 67.8% (OR) monomer reduction, and the aggregate level increased by 44.4% for BS and 67.8% for OR. Figure 5 shows the results and overlay chromatograms for pH 4 stress. Basic pH 9 incubation induced near-identical aggregation increases (BS: 0.97%; OR: 0.95%) with corresponding 1.0% monomer decreases in both samples, demonstrating comparable alkaline stability. A slope comparison of monomer and aggregate linear curves revealed significant differences under acidic pH stress (*p* < 0.0001) but no significant variation in basic conditions, demonstrating pH-dependent degradation kinetics. While the aggregation patterns of the BS and OR were similar under basic conditions, significant differences were found under acidic pH stress, with the OR showing a greater decrease in monomer levels. This suggests that differences in manufacturing processes or glycosylation profiles result in varying structural stability between biosimilar and originator products [18]. Such differences highlight the importance of further characterization to guarantee biosimilarity in terms of degradation pathways and stability profiles.

Under acidic pH stress, the main and acidic charge variants decreased, and the basic charge variant amounts increased in the BS and OR samples. In the BS sample, the main charge variant decreased by 8.1% and the acidic charge variant by 2.4%, while the basic charge variant increased by 10.5%. Under the same stress conditions, in the OR sample, the main charge variant decreased by 6.6% and the acidic variant by 8.4%, while the basic charge variant increased by 15.0%. This indicates that basic charge variants emerged under acidic pH conditions, showing that the mAb acquired more basic properties. These results demonstrate a transition from main and acidic charge variants to basic charge variants in both the BS and OR samples. Conversely, under basic pH stress, acidic charge variants increased by 3.9% in BS and 4.1% in OR, with no significant changes observed in the basic charge variants. Overall, the findings indicate that most main charge variants in the BS and OR samples were transformed into acidic charge variants.

Under acidic pH stress, BS/OR showed LC + HC decreases (2.3%/2.2%) and IgG reductions (0.98%/1.2%) versus the control. Basic stress caused LC + HC declines (1.7%/2.0%) and IgG decreases (0.60%/0.77%).

In most cases, mAb instability results from aggregate formation. Aggregates have either reduced or no biological activity and may be immunogenic [38]. IgG aggregates when exposed to low pH during purification processes such as residual Protein A chromatography and low pH virus inactivation [39,40]. The observed changes in charge variant profiles further support these findings. Since pH 4 was the harshest condition, relative potencies were evaluated only for samples under this condition. BS and OR potency equally declined to 87.6% of the control. The increased aggregation of mAbs, which are capable of eliciting an immunological response, as observed in the pH-altered results, supports the decrease in antibody activity demonstrated by the complement assay analysis. The increased conversion of main charge variants into basic or acidic forms under pH 4 stress conditions may also influence receptor binding and bioactivity.

For the development of biosimilars, our findings reinforce the need for rigorous forced degradation studies to assess stability comparability. Future research should explore the interaction between formulation components such as excipients and stabilizers in reducing pH-induced degradation. Overall, this study highlights the importance of pH stability in ensuring mAb efficacy and safety.

### 2.4. Freeze/Thaw Stress

During handling and transport, known as freeze/thaw (F/T) cycles, mAb products often undergo stress conditions. These cycles can subject mAbs to various stresses that may lead to aggregation. The stresses include the cold denaturation of the mAb solution, changes in pH due to buffer crystallization, and the disruption of the protein conformation caused by the ice–solution interface [41,42]. In addition, the increased salt concentration resulting from freezing can reduce repulsion between protein molecules, which may result in intermolecular interactions and aggregation [36,43,44].

While the formation of different aggregates and fragments is the primary mechanism of F/T stress, chemical degradation is not the main pathway of concern. Consequently, the most frequently used analytical techniques in F/T studies focus on purity and size analysis [35,38].

**Figure 6:**
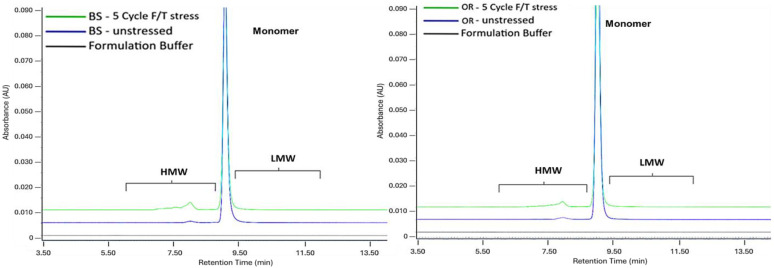
SE-UPLC chromatograms of BS (**left**) and OR (**right**) subjected to freeze and thaw stress for 5 cycles. Samples were analyzed at a concentration of 2.5 mg/mL.

After undergoing five cycles of F/T stress, monomer levels decreased by 6.02% for BS and 6.0% for OR, while aggregate levels increased by 6.0% and 6.1% compared to the control samples. Figure 6 presents the overlay chromatograms. No significant differences were observed when comparing the levels of charge variants in the BS and OR samples with their control samples. The main charge variants decreased (BS:2.0%; OR:1.6%) while acidic variants increased (BS:1.6%; OR:1.2%) and basic variants rose slightly (BS:0.42%; OR:0.40%). These observations are consistent with previous reports that F/T stress has a limited effect on charge heterogeneity compared to other stress conditions, such as oxidation or deamidation [18,42].

To better characterize the nature of the aggregates formed, CE-SDS analyses were conducted to differentiate between covalently and noncovalently linked aggregates. The LC + HC levels of BS and OR samples decreased by 0.95% and 0.94%, respectively, compared to the control samples. Furthermore, the %IgG levels in F/T stress samples decreased by 0.51% for BS and 0.66% for OR. The CE-SDS results confirmed that the aggregates were primarily noncovalent. This finding aligns with a previous study that examined humanized IgG1 mAbs exposed to thermal and freeze/thaw (F/T) stress, which showed varying aggregation profiles. Specifically, thermal stress resulted in the formation of partially covalent aggregates, while F/T stress led to the creation of noncovalent aggregates [43,45]. This indicates that although both stress conditions can trigger aggregation, the underlying mechanisms of aggregate formation differ. F/T-induced aggregation is likely driven by physical interactions rather than chemical modifications.

Comparable aggregation profiles and charge variant distributions between BS and OR indicate that they exhibit similar stability and degradation behavior under F/T stress. This is critical for establishing biosimilarity, as differences in aggregation propensity may affect immunogenicity and therapeutic efficacy. The relatively low aggregation levels observed in the BS and OR samples suggest that the formulation is robust against F/T-induced instability, reducing potential risks in clinical use.

### 2.5. Oxidation Stress

Upon exposure to substances such as free radicals and oxygen, mAbs readily oxidize [46,47]. Throughout their production and storage, oxidation can affect residues such as methionine, tryptophan, and tyrosine. Therefore, monitoring oxidation is essential for quality control in mAbs [48,49]. Exposing mAbs to oxidative stress tests how sensitive residues affect degradation [50,51]. Oxidation in the CDR can impair the binding of antibodies to Fc receptors and antigens, thereby affecting mAb stability and half-life. The oxidation of Met residues can also cause increased immunogenicity [46,51,52].

BioPhorum’s recent survey revealed that most companies utilize oxidation stress to gain insights into mAb degradation mechanisms. The majority utilize hydrogen peroxide (H_2_O_2_) as an oxidizing agent, with concentrations ranging from 0.001% *v*/*v* to 0.5% *v*/*v*. The exposure duration varies from a few hours up to three days. During oxidation stress, the preferred incubation temperature is 25 °C, and changing the buffer after the incubation period is necessary [22].

In this study, exposure to 0.5% H_2_O_2_ (*v*/*v*) oxidative stress reduced monomers (BS: 1.5%; OR: 1.3%), with near-equivalent aggregate increases. Under harsher oxidative stress (3.0% H_2_O_2_, 24 h, 25 °C), aggregates increased more significantly (BS: 2.0%; OR: 3.0%) [53].

**Figure 7:**
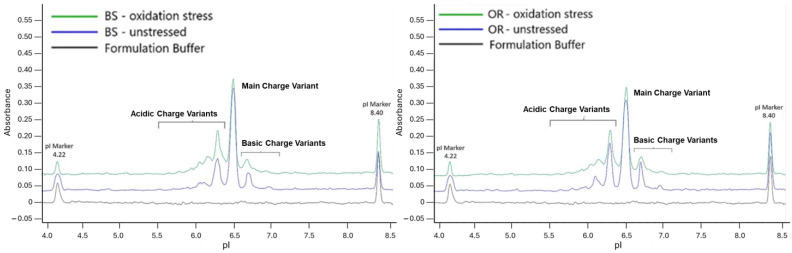
icIEF electropherograms of BS (**left**) and OR (**right**) subjected to oxidation stress for 24 h. Samples were analyzed at a concentration of 3.0 mg/mL.

The main peak for BS and OR decreased by 12.1% and 12.3%, respectively, according to the charge variant characterization. The observed decrease in the main peak is directly related to the increase in the acidic peak levels. Furthermore, one study revealed that oxidation in the F(ab’)_2_ region led to the formation of acidic variants, while oxidation in the Fc region produced basic variants [54]. These charge modifications could impact the binding affinity and stability of the mAb, influencing its efficacy and safety. Figure 7 presents the overlay electropherograms.

The nrCE-SDS analysis clearly revealed that degradation, rather than aggregation, occurred in the BS and OR samples due to oxidative stress. Relative to the control, the IgG level decreased by 6.6% for the BS and 6.5% for the OR samples after exposure to oxidative stress. LC + HC levels also dropped (BS: 0.89%, OR: 0.49%) versus the control. The significant decrease in IgG% and LC + HC% levels under oxidative stress highlights the sensitivity of mAbs to oxidation, leading to possible degradation. Previous studies with lower H_2_O_2_ concentrations (0.02%) reported minimal monomer loss, whereas the higher concentration used here (0.5%) caused greater degradation. These findings are consistent with studies showing that oxidative stress can disrupt the structural integrity of mAbs, leading to aggregation and functional loss [37,55,56].

Oxidative modifications can affect antigen binding, interactions with Fc receptors, and complement activation, ultimately influencing the therapeutic performance of mAbs. Since aggregates are linked to increased immunogenicity, the observed aggregation caused by oxidative stress raises concerns about patient safety. The biological activity of the BS and OR samples decreased upon exposure to oxidation stress. The relative potency results for BS decreased to 86.3% and for OR to 74.7%. Despite increased acidic charge variants and decreased IgG%, the complement assay results consistently fell within the acceptable range. Notably, reducing the risk of oxidative degradation will require optimizing long-term storage and handling conditions. In an oxidative stress study using adalimumab and its biosimilar, exposure to H_2_O_2_ increased the formation of aggregates, which negatively affected biological activity [53].

Despite their effectiveness in characterizing degradation pathways, methods such as UPLC, CE-SDS, and charge variant analysis have inherent limitations, including the inability to fully discriminate between covalent and noncovalent aggregates, potential interference from formulation components, and sensitivity limitations for detecting subtle changes. These challenges highlight the need for additional methodological improvements as well as multiple analytical techniques to enable a more comprehensive assessment of the stability of biosimilar mAbs.

## 3. Materials and Methods

All chemicals and reagents were of analytical grade. Materials used for analyses were supplied by local vendors. Monosodium phosphate, sodium azide, iodoacetamide, citric acid monohydrate, and sodium bicarbonate were purchased from Sigma Aldrich (Darmstadt, Germany). Merck (Darmstadt, Germany) supplied 2-mercaptoethanol, sodium chloride, hydrogen peroxide, and sodium hydroxide. Wide-range ampholytes were purchased from Pharmalyte (Cytiva, Uppsala, Sweden) and narrow-range ampholytes from Bio-Rad (Hercules, CA, USA). Isoelectric point (pI) markers 4.22 and 8.40 were purchased from Protein Simple (San Jose, CA, USA). Chromatograms and electropherograms were collected and processed with Empower 3 software. (Waters, Milford, MA, USA). GraphPad Prism 9.1.3 (GraphPad Software, Inc., San Diego, CA, USA) software was used for the statistical analysis.

### 3.1. Sample Details

Both the originator (abbreviated OR) and biosimilar (abbreviated BS) products are humanized IgG2/4 kappa antibodies. The biosimilar candidate was developed, manufactured, purified, and concentrated by Turgut Ilacları A.S. (Kocaeli, Türkiye) in Türkiye. The originator is manufactured by Alexion and marketed under the brand name Soliris. The contents of the formulation buffer were the same for both products, with a final target protein concentration of 10 mg/mL. Two different batches of the originator were purchased from the local pharmacy and stored at 5.0 ± 3.0 °C, as per the manufacturer’s instructions, until use.

### 3.2. Sample Preparation

Samples underwent various stress types to assess degradation using various analytical methods. Forced degradation studies involved thermal, agitation, pH (acid and base), freeze/thaw, and oxidation stress. All stress conditions were applied to the biosimilar mAb (BS) and two originator product samples (OR). Formulation buffer (FB) without mAb was used as blank control and the same stress conditions as samples with mAb were applied in every stress condition. All samples subjected to stress conditions were stored at 5.0 ± 3.0 °C until analysis. Following the incubation period, the samples exposed to pH and oxidation stresses were buffer exchanged back to their FB. Buffer exchange was performed after the pH and oxidation stress treatments to remove residual reactants from the stress conditions and to ensure that the subsequent analytical measurements were performed in a controlled environment.

#### 3.2.1. Thermal Stress

The thermal stress was induced by incubating the samples in a Thermomixer (Eppendorf, Montesson, France) at two different temperatures, 37 °C and 50 °C, without agitation and humidity control. Samples were collected for analysis on days 3, 7, and 14 to assess the impact of thermal stress over time. To ensure the integrity of the samples, the tube caps were securely sealed to prevent evaporation.

#### 3.2.2. Agitation Stress

To subject the samples to agitation stress, they were placed in a Compact Digital Rocker (Thermo Scientific, Waltham, MA, USA) and agitated at 400 rpm at 25 ± 2 °C room temperature (RT). Samples were pulled after 24 and 72 h.

#### 3.2.3. pH Stress

To induce acidic pH stress, the samples were titrated to pH 4.0 using 1 M hydrochloric acid and then placed at RT for 72 h. For basic pH stress, the samples were titrated with 1 M Tris to pH 9.0 and incubated at RT for 72 h.

#### 3.2.4. Freeze/Thaw Stress

In the freeze/thaw (F/T) studies, the samples were initially cooled from 5 ± 3 °C to ≤−65 °C, then kept at ≤−65 °C for two hours and subsequently thawed to 5 ± 3 °C, followed by a two-hour holding period for each cycle. After completing five F/T cycles, the samples were stored at 5 ± 3 °C until analysis.

#### 3.2.5. Oxidation Stress

To induce oxidative stress in the samples, hydrogen peroxide was added to each sample to achieve a final concentration of 0.5% (*v*/*v*). Subsequently, the samples were left to incubate at RT for 24 h to observe the effects of oxidative stress on the samples.

### 3.3. Size Exclusion Ultra-Performance Liquid Chromatography (SE-UPLC)

The separation of aggregates, monomers, and fragments was conducted using an Acquity H-Class Bio UPLC^®^ system featuring a UV detector equipped with a titanium flow cell in conjunction with an Acquity UPLC^®^ Protein BEH column (200 Å, 1.7 μm, 4.6 mm × 300 mm). An isocratic flow of the mobile phase, composed of 20 mM sodium phosphate with 188 mM sodium chloride at pH 7.4, was employed for separation at a flow rate of 0.25 mL/min. Each sample, diluted with the mobile phase, was injected (6.25 µg) into the column. UV detection was conducted at 280 nm for a 20 min run time. The data acquisition, equipment control, and data processing were performed using Empower 3 software (Waters, Milford, MA, USA).

### 3.4. Imaged Capillary Isoelectric Focusing (icIEF)

The analysis of charge heterogeneity and the determination of the isoelectric point (pI) of the samples were conducted using the Protein Simple iCE3 Capillary IEF system with a (50 μm i.d. × 20 cm) fluorocarbon-coated capillary. The samples, diluted to a concentration of 3.0 mg/mL in water, were combined with a master mix containing 2% Pharmalyte ampholytes 3–10, 1% Biolyte ampholytes pH 4–6, 1% Biolyte ampholytes pH 6–8, 0.35% methylcellulose, pI 4.22 and pI 8.40 marker (0.5 *v*/*v*%), and additives (8 M urea, 125 mM iminodiasetic acid, and 500 mM arginine). Subsequently, the sample mixture was injected into the cartridge and pre-focused at 1500 V for 2 min, followed by focusing at 3000 V for 10 min. Data acquisition was performed at 280 nm using iCE3 CFR software version 5.0.0.5934, and the resulting electropherograms were imported into Empower 3 (Waters, Milford, MA, USA) software for comprehensive data analysis.

### 3.5. Capillary Electrophoresis-Sodium Dodecyl Sulfate (CE-SDS)

The analysis utilized the Beckman Coulter (Brea, CA, USA) PA 800 Plus system with a photodiode array detector to assess total purity and detect impurities. The separation process utilized a bare-fused silica capillary (50 µm × 67 cm) and SDS-Mw gel buffer (pH 8, 0.2% SDS). For reduced CE-SDS (rCE-SDS), samples at a concentration of 1.0 mg/mL were combined with SDS sample buffer (100 mM Tris-HCl, pH 9.0, 1% SDS) and reduced with βME, followed by heating to 70 °C for 10 min. For non-reduced CE-SDS (nrCE-SDS), samples were prepared with 250 mM iodoacetamide and SDS sample buffer and then heated to 70 °C for 10 min. Each sample contained a total of 100 µg of protein. The samples were injected into the capillary at 5 kV in reverse polarity for 20 s, and separation was conducted at 15 kV for 40 min. Data were captured at 220 nm with a reference channel set to 350 ± 10 nm, using the 32 Karat software (Beckman Coulter), and analyzed using Empower 3 (Waters, Milford, MA, USA).

### 3.6. Complement Assay

The enzyme-linked immunosorbent assay employing the complement classical pathway was utilized to evaluate the potency of the samples relative to a reference standard (WIESLAB Complement Classical Pathway COMPLCP310). The samples underwent dilution and were subsequently combined with normal human serum, known to contain the complement pathway under specified immunoassay conditions. A concentration gradient of the samples was produced and added to a plate designed to selectively activate the pathway. Then, a labeled antibody specific to a neoepitope of the terminal complement complex was added. Following that, a p-Nitrophenyl Phosphate (pNPP) substrate was added, and it was converted to a colorimetric product based on the amount of bound labeled antibody with Spectramax i3X multi-mode microplate reader (Molecular Devices, San Jose, CA, USA). The concentration of this product was measured at 405 nm and used to determine the relative potency of the material. The system control, data acquisition, and analysis were performed using Softmax Pro software version 7.1.1.

### 3.7. Statistical Analysis

One-way analysis of variance Dunn’s multiple comparisons test was used to compare the differences between biosimilar (BS) and originator (OR) samples. The results are expressed as the mean ± SD, with a significance level of *p* < 0.05. The statistical analysis was performed using GraphPad Prism Software version 9.0.

## 4. Conclusions

This study evaluated the similarity between BS and OR regarding their degradation profiles. Acidic and basic charged variants, aggregates, and biological activities were assessed using high-resolution physicochemical and biological characterization methods. The results indicated that the BS is analytically similar to the OR in key parameters related to efficacy and safety, even under various stress conditions. In addition, a significant correlation was found between the results of physicochemical analysis and complement assay analysis, emphasizing the effect of stress conditions on the overall activity of the drug product. The study also validated analytical methods for stress-exposed BS product evaluation regarding production, stability, and quality attributes, revealing that degradation products formed under these conditions can be identified and quantitatively compared. Overall, the forced degradation studies evaluated the structural integrity and biological activity of the mAb, provided insights into product stability, clarified degradation pathways, and identified critical quality attributes. This study contributes to the existing literature on BS development and supports future research aimed at advancing biopharmaceutical drug candidates.

## Figures and Tables

**Table 1:** SE-UPLC, CE-SDS, and icIEF results of untreated (control) and treated BS samples. Results are given as area %, mean ± SD. *p*-values were obtained from Dunn’s multiple comparison tests of treated samples with controls at each time point.

		SE-UPLC		CE-SDS		icIEF		
		Monomer	HMW	LC + HC	IgG	Main Variant	Acidic Variants	Basic Variants
	**BS control**	97.9 ± 0.01	1.2 ± 0.01	99.4 ± 0.04	97.8 ± 0.2	59.6 ± 0.24	28.6 ± 0.30	11.8 ± 0.53
**Thermal Stress**	37 °C, 3 d	97.1 ± 0.01	2.0 ± 0.01	98.3 ± 0.00	97.6 ± 0.04	58.1 ± 0.02	30.8 ± 0.05	11.1 ± 0.07
	*p*-value	>0.99	0.49	0.98	>0.99	>0.99	>0.99	>0.99
	37 °C, 7 d	97.0 ± 0.01	2.2 ± 0.01	97.9 ± 0.05	97.5 ± 0.02	55.6 ± 0.11	33.8 ± 0.01	10.6 ± 0.11
	*p*-value	0.22	0.22	0.49	>0.99	>0.99	>0.99	0.77
	37 °C, 14 d	96.6 ± 0.01	2.4 ± 0.01	97.2 ± 0.04	96.4 ± 0.02	49.8 ± 0.18	40.1 ± 0.03	10.1 ± 0.16
	*p*-value	0.01 *	0.01 *	0.01 *	0.361	0.286	0.066	0.458
	50 °C, 3 d	94.6 ± 0.01	4.4 ± 0.01	97.2 ± 0.16	97.6 ± 0.03	49.1 ± 0.04	41.4 ± 0.14	9.5 ± 0.18
	*p*-value	>0.99	>0.99	>0.99	>0.99	>0.99	>0.99	>0.99
	50 °C, 7 d	92.7 ± 0.01	6.2 ± 0.01	96.2 ± 0.07	97.0 ± 0.04	37.6 ± 0.26	53.8 ± 0.12	8.6 ± 0.14
	*p*-value	0.98	0.98	0.38	>0.99	0.54	0.54	0.62
	50 °C, 14 d	89.6 ± 0.02	9.0 ± 0.02	92.2 ± 0.50	96.4 ± 0.06	25.1 ± 0.01	67.6 ± 0.28	7.4 ± 0.29
	*p*-value	0.09	0.09	0.01 *	0.12	0.07	0.07	0.34
**Agitation Stress**	24 h	97.3 ± 0.01	1.9 ± 0.01	98.4 ± 0.03	97.4 ± 0.07	57.2 ± 0.16	30.4 ± 0.02	12.3 ± 0.13
	*p*-value	0.08	0.08	0.49	>0.99	>0.99	>0.99	>0.99
	72 h	97.3 ± 0.02	1.9 ± 0.01	98.3 ± 0.09	97.2 ± 0.02	56.0 ± 0.21	31.3 ± 0.30	12.7 ± 0.09
	*p*-value	0.02 *	0.02 *	0.36	0.54	>0.99	>0.99	>0.99
**pH Stress**	pH 4, 72 h	53.6 ± 0.02	45.6 ± 0.03	97.1 ± 0.08	96.8 ± 0.04	51.6 ± 0.15	26.2 ± 0.04	22.2 ± 0.18
	*p*-value	0.98	0.98	0.08	0.08	0.19	0.53	0.19
	pH 9, 72 h	96.9 ± 0.04	2.2 ± 0.02	97.7 ± 0.12	97.2 ± 0.02	55.9 ± 0.08	32.5 ± 0.51	11.6 ± 0.43
	*p*-value	0.08	0.08	0.08	0.41	>0.99	>0.99	>0.99
**Freeze/Thaw Stress**	F & T	91.9 ± 0.01	7.2 ± 0.01	98.4 ± 0.23	97.3 ± 0.06	57.6 ± 0.01	30.2 ± 0.03	12.2 ± 0.04
	*p*-value	0.08	0.08	0.22	0.50	>0.99	>0.99	>0.99
**Oxidation Stress**	24 h	96.4 ± 0.01	2.8 ± 0.02	98.5 ± 0.06	91.2 ± 0.07	47.5 ± 0.27	40.7 ± 0.11	12.0 ± 0.16
	*p*-value	0.08	0.08	0.13	0.08	0.19	0.19	>0.99

* Statistically significant differences (*p*-value < 0.05) between control and stress condition were marked.

**Table 2:** SE-UPLC, CE-SDS, and icIEF results of untreated (control) and treated OR samples. Results are given as area %, mean ± SD. *p*-values were obtained from Dunn’s multiple comparison tests of treated samples with controls at each time point. * Statistically significant differences (*p*-value < 0.05) marked.

		SE-UPLC		CE-SDS		icIEF		
		Monomer	HMW	LC + HC	IgG	Main Variant	Acidic Variants	Basic Variants
	**OR control**	98.1 ± 0.02	1.0 ± 0.02	98.5 ± 0.02	98.6 ± 0.03	52.7 ± 0.21	34.6 ± 0.29	12.7 ± 0.23
**Thermal Stress**	37 °C, 3 d	97.3 ± 0.01	1.8 ± 0.02	98.0 ± 0.62	98.5 ± 0.14	51.2 ± 0.11	36.6 ± 0.16	12.3 ± 0.11
	*p*-value	0.8308	0.83	>0.99	>0.99	>0.99	>0.99	>0.99
	37 °C, 7 d	97.1 ± 0.01	1.9 ± 0.01	97.3 ± 0.09	97.8 ± 0.10	49.3 ± 0.13	39.2 ± 0.08	11.5 ± 0.09
	*p*-value	0.02 *	0.08	0.04 *	0.49	0.27	0.66	0.41
	37 °C, 14 d	96.9 ± 0.01	2.3 ± 0.04	96.7 ± 0.06	97.5 ± 0.14	43.5 ± 0.25	45.4 ± 0.28	11.1 ± 0.08
	*p*-value	<0.0001 *	<0.0001 *	0.0002 *	0.001 *	0.03 *	0.03 *	0.01 *
	50 °C, 3 d	94.4 ± 0.01	4.6 ± 0.02	96.3 ± 0.20	98.1 ± 0.13	43.6 ± 0.56	45.0 ± 0.43	11.4 ± 0.27
	*p*-value	0.2892	0.2902	0.6007	>0.99	>0.99	>0.99	>0.99
	50 °C, 7 d	92.1 ± 0.01	6.9 ± 0.02	94.2 ± 0.08	97.4 ± 0.17	32.4 ± 0.31	58.5 ± 0.44	9.0 ± 0.16
	*p*-value	0.003 *	0.003 *	0.02 *	0.02 *	0.10	0.10	0.10
	50 °C, 14 d	86.4 ± 0.01	12.2 ± 0.01	90.6 ± 0.21	96.8 ± 0.07	19.6 ± 0.15	73.3 ± 0.14	7.1 ± 0.04
	*p*-value	<0.0001 *	<0.0001 *	<0.0001 *	<0.0001 *	0.002 *	0.002 *	0.0004 *
**Agitation Stress**	24 h	97.6 ± 0.01	1.5 ± 0.02	97.6 ± 0.31	98.0 ± 0.05	50.4 ± 0.04	36.3 ± 0.08	13.3 ± 0.06
	*p*-value	0.19	0.14	0.01 *	0.60	>0.99	>0.99	0.66
	72 h	97.5 ± 0.01	1.6 ± 0.01	97.5 ± 0.34	97.8 ± 0.07	48.6 ± 0.20	37.7 ± 0.04	13.7 ± 0.23
	*p*-value	0.005 *	0.01 *	0.003 *	0.02 *	0.1363	0.1359	0.0571
**pH Stress**	pH 4, 72 h	30.3 ± 0.02	68.8 ± 0.01	96.3 ± 0.06	97.4 ± 0.09	46.2 ± 0.13	26.2 ± 0.18	27.6 ± 0.07
	*p*-value	0.0002 *	0.0002 *	0.01 *	0.01 *	0.04 *	0.01 *	0.04 *
	pH 9, 72 h	97.0 ± 0.01	2.0 ± 0.01	96.6 ± 0.28	97.8 ± 0.05	48.7 ± 0.14	38.7 ± 0.18	12.6 ± 0.13
	*p*-value	0.01 *	0.01 *	0.01 *	0.04 *	0.23	0.23	>0.99
**Freeze/Thaw Stress**	F & T	92.1 ± 0.01	7.1 ± 0.01	97.6 ± 0.17	97.9 ± 0.06	51.2 ± 0.16	35.8 ± 0.14	13.1 ± 0.03
	*p*-value	0.01 *	0.01 *	0.02 *	0.05 *	0.23	0.23	0.23
**Oxidation Stress**	24 h	96.8 ± 0.19	2.4 ± 0.21	98.0 ± 0.42	92.1 ± 0.14	40.5 ± 0.17	46.3 ± 0.25	13.1 ± 0.09
	*p*-value	0.01 *	0.01 *	0.0216 *	0.01 *	0.04 *	0.04 *	0.23

* Statistically significant differences (*p*-value < 0.05) between control and stress condition were marked.

## Data Availability

Data are contained within the article.

## Abbreviations

The following abbreviations are used in this manuscript:

BS	Biosimilar monoclonal antibody
CE-SDS	Capillary Electrophoresis Sodium Dodecyl Sulfate
F/T	Freeze/thaw
HC	Heavy Chain
HMW	High Molecular Weight
icIEF	Imaged Capillary Isoelectric Focusing
LC	Light Chain
LMW	Light Molecular Weight
mAb	Monoclonal antibody
nrCE-SDS	Non-Reduced Capillary Electrophoresis-Sodium Dodecyl Sulfate
OR	Originator Monoclonal Antibody
rCE-SDS	Reduced Capillary Electrophoresis-Sodium Dodecyl Sulfate
SD	Standard Deviation
SE-UPLC	Size Exclusion Ultra High-Performance Liquid Chromatography
